# Efficacy, Durability, and Safety of VYC‐17.5L Hyaluronic Acid Dermal Filler for Facial Enhancement: A Systematic Review and Single‐Arm Meta‐Analysis

**DOI:** 10.1111/jocd.71233

**Published:** 2026-10-11

**Authors:** Sadiq M. Altbali, Ibrahim Yassin, Muhammad Saif ur Rehman, Yara Alswaiti, Mahmoud Ashraf Hussein, Manar Hamdy, Sohaila Mohamed Mohamed Abdelbar, Mohammed Aljameie, Jawaria Firdous, Alaa Mohammed Oudah, Mohammad Qaq, Eathar Aljubori, Khalid Mohammed Al‐Dhayani, Aya Ahmed Shimal

**Affiliations:** ^1^ Faculty of Medicine Ibb University Ibb Yemen; ^2^ Faculty of Medicine Beni‐suef University Beni‐suef Egypt; ^3^ Khyber Medical University Peshawar Pakistan; ^4^ Faculty of Medicine Hashemite University Zarqa Jordan; ^5^ Faculty of Medicine Capital University Cairo Egypt; ^6^ Faculty of Medicine Kafrelsheikh University Kafr El‐Sheikh Egypt; ^7^ Kasralainy Medical Hospitals Cairo University Cairo Egypt; ^8^ Hammurabi College of Medicine University of Babylon Babil Iraq; ^9^ Faisalabad Medical University/Punjab Medical College Faisalabad Pakistan; ^10^ Alkindy College of Medicine University of Baghdad Baghdad Iraq; ^11^ Faculty of Medicine Al‐Quds University Jerusalem Palestine; ^12^ College of Medicine Kufa University Najaf Iraq; ^13^ Faculty of Medicine Amran University Amran Yemen; ^14^ College of Medicine University of Baghdad Baghdad Iraq

## Abstract

**Background:**

Facial aesthetics encompasses a variety of techniques to revitalize the face through aesthetic medicine. Satisfaction with one's face is important to well‐being, leading people to seek to maintain their youthful appearance throughout their lives. Accordingly, many techniques have been developed for facial rejuvenation. VYC‐17.5L (Volift/Vollure) is a hyaluronic acid–based dermal filler utilized for facial enhancement; however, evidence regarding its long‐term efficacy and safety remains inconsistent.

**Methods:**

Following PRISMA guidelines, a systematic search was performed across PubMed, Scopus, Web of Science, ScienceDirect, Embase via Ovid, and Cochrane Library to identify studies evaluating VYC‐17.5L (Volift/Vollure) hyaluronic acid dermal fillers for facial aesthetic enhancement. Eligible studies included randomized controlled trials, prospective cohort studies, retrospective observational studies, and case series with ≥ 10 patients. Risk of bias was assessed using the Cochrane RoB 2 tool for randomized studies and ROBINS‐I for non‐randomized studies. Single‐arm meta‐analysis was performed to evaluate aesthetic outcomes (GAIS, FACE‐Q, NLFSS, WSRS, and responder rates) and safety outcomes.

**Results:**

Eleven studies comprising 967 participants were included. Clinician‐assessed GAIS demonstrated a time‐dependent decline, reaching its peak at 3 months (87%–90%) and decreasing to (24%–33%) at 12 months. Patient‐reported GAIS responder rates remained high through 6 months (85%–95%) but declined to 62% at 9 months and 53% at 12 months. FACE‐Q responder rates remained high across the available 12‐month follow‐up. NLFSS responder rates remained consistently high (≥ 89%) up to 6 months, indicating sustained improvement in nasolabial fold severity. Adverse events were predominantly mild and transient; clinician‐reported frequencies were low, whereas patient‐reported frequencies were considerably higher.

**Conclusion:**

VYC‐17.5L demonstrates favorable short‐term efficacy and safety for facial rejuvenation, with high patient satisfaction. Objective aesthetic improvements decline over time, highlighting the time‐limited nature of treatment effects and the importance of appropriate patient counseling.

**Trial Registration:** PROSPERO number: CRD420261279046

## 1 Introduction

Skin aging is a multifactorial process demonstrated by loosening and ptosis of the soft tissue of the face and neck due to gravity and loss of the distinct contour of the neck and jawline. These changes result in the characteristic features of facial aging. Since the introduction of filler injection procedures for cosmetic and reconstructive purposes, it has become widely popular with patients seeking treatment for signs of aging with a quick return to daily life [1, 2, 3].

Facial aesthetics encompasses a variety of techniques to revitalize the face through aesthetic medicine. Satisfaction with one's face is important to well‐being, leading people to seek to maintain their youthful appearance throughout their lives. Accordingly, many techniques have been developed for facial rejuvenation, such as neuromodulators, laser treatment, bleaching, chemical peeling, and suturing [4].

However, each technique addresses the face and time‐related changes in a specific area; facial fillers are more flexible in modifying the treatment area to meet patients' needs. Flexible regeneration requires a deeper understanding of facial anatomy and the full interplay of life sciences and art [5].

Aesthetic beautification anticipates the patient's needs, and an analysis of the face improves satisfaction through newly arranged projections, a rejuvenating yet healthy look, and vibrant emotional expression. The development of intelligent facial analysis reflects these requirements and concepts for professional facial assessment. Although skin aging is the trend which has been maintained for many decades, there are concerns associated with using fillers in scars and wound healing processes [6].

HA is a natural polysaccharide polymer composed of polymeric disaccharides of D‐glucuronic acid and *N*‐acetyl‐D‐glucosamine mixture [7]. This disaccharide polymer is abundantly present in humans (~15 g in a 70 kg man) and is detected in the umbilical cord, joints, vitreous humor, skin, and connective tissue [8].

When HA was first used in ophthalmic surgery during the 1970s, it attracted the direct attention of researchers and has been further explored in indifferent and far away medical fields. HA shows high hygroscopicity, rheology, viscoelasticity, and water‐holding capacity, which have propelled its application in aesthetic dermatology, expecting to regulate skin hydration, stimulate collagen production, increase elasticity, slow down wrinkle formation, and achieve an antiaging and smoothing effect [9, 10].

It interacts with specific proteins like CD44, RHAMM, ICAM‐1, and LYVE1. Because CD44 is the most prominent receptor, HA binding to CD44 on keratinocytes activates signaling pathways that influence cell differentiation and motility. Additionally, RHAMM facilitates cell migration and adhesion during inflammation. Furthermore, HA's water‐retaining properties make it a popular ingredient in cosmetic products, where it is found in various forms such as creams, gels, and serums. Research indicates that HA supplementation can improve skin hydration, promote collagen production, reduce wrinkles, and contribute to facial rejuvenation [9, 10].

Moreover, chemical modification of HA molecules has paved the way for HA‐based formulations, holding diverse applications in wound coverage, tissue engineering, and regenerative medicine.

VYC‐17.5L—marketed as Juvederm Volift in Europe and Juvederm Vollure in the US—is a hyaluronic acid (HA) dermal filler formulated using Vycross technology. This proprietary platform combines low‐ and high‐molecular‐weight HA to maximize tissue integration, longevity, and aesthetic naturalness by increasing the cross‐linking efficiency of the HA chains. VYC‐17.5L (Volift/Vollure) offers a versatile middle‐ground concentration (17.5 mg/mL). It is designed for deep nasolabial folds, perioral rejuvenation, and lip augmentation, offering a soothing, natural effect with a low risk of swelling or nodules. VYC‐17.5L formulations typically include lidocaine to minimize pain during administration and demonstrate excellent responses following injection. Clinical studies have suggested that VYC‐17.5L may provide sustained aesthetic improvement with high patient satisfaction, but reported outcomes vary across populations, treatment areas, and study designs [9, 10].

Despite the increasing use of VYC‐17.5L, there is currently no comprehensive synthesis of available evidence regarding its overall aesthetic effectiveness and safety profile. Existing literature is fragmented, comprising randomized controlled trials, observational cohorts, and case series with heterogeneous outcome measures, including the Global Aesthetic Improvement Scale (GAIS), NLFSS, WSRS, and patient‐reported satisfaction scores [11].

The lack of pooled data limits the ability of clinicians and patients to make evidence‐based decisions regarding the choice of filler for facial enhancement. This systematic review and meta‐analysis aims to critically evaluate the aesthetic outcomes, patient satisfaction, durability, and safety of VYC‐17.5L across published clinical studies to provide a comprehensive and clinically relevant evaluation of VYC‐17.5L's clinical performance and guide future practice in facial aesthetic treatments.

## 2 Methods

This systematic review and single‐arm meta‐analysis was conducted according to the PRISMA guideline (Preferred Reporting Items for Systematic Reviews and Meta‐Analyses), in alignment with the Cochrane Handbook for Systematic Reviews of Interventions [12], and was prospectively registered on PROSPERO (CRD420261279046).

### 2.1 Eligibility Criteria

The research question was structured according to the population (P), intervention (I), and outcome (O) framework. However, because most included studies were single‐arm studies, a comparator was not required for eligibility and was considered when available in comparative studies. The population (P) represented adults (≥ 18 years) undergoing facial aesthetic treatment. The intervention (I) represented injection with VYC‐17.5L (Volift/Vollure) hyaluronic acid dermal fillers with a concentration of 17.5 mg/mL. The comparator (C) was applicable only when reported in comparative studies. The outcomes (O) included aesthetic improvement measured by GAIS (Global Aesthetic Improvement Scale), NLFSS (Nasolabial Fold Severity Scale), patient satisfaction scores, and Investigator Satisfaction Scales. Secondary outcomes included responder rate (≥ 1‐point improvement), WSRS (Wrinkle Severity Rating Scale), incidence of adverse events (e.g., swelling, pain after injection, tenderness, bruising, discoloration, induration, itching, lumps, redness), and retreatment rates.

Studies were included if they met the following criteria: (1) human adults (≥ 18 years) undergoing facial aesthetic treatments; (2) use of VYC‐17.5L (Volift/Vollure) hyaluronic acid dermal fillers for volume restoration, wrinkle correction, or facial enhancement; (3) randomized controlled trials (RCTs), prospective cohort studies, retrospective observational studies, or case series with ≥ 10 patients; (4) reported aesthetic outcomes including GAIS, NLFSS, patient satisfaction, longevity or duration of effect, and adverse events; and (5) articles published in English.

Articles were excluded if they were: (1) studies using other HA fillers without clearly reporting separate outcomes for VYC‐17.5L (Volift/Vollure); (2) case reports with < 10 patients, review articles, editorials, letters, or preclinical studies (animal or in vitro); (3) studies not reporting objective or subjective aesthetic outcomes; (4) studies combining multiple fillers or treatments without separate outcome data for VYC‐17.5L; or (5) multiple publications using the same patient population without new or distinct analysis.

### 2.2 Search Strategy for Databases

A systematic review of the published literature was conducted in accordance with the Preferred Reporting Items for Systematic Reviews and Meta‐Analyses (PRISMA) guidelines. The search strategy was developed by one reviewer and reviewed by a second reviewer. The following databases were searched: PubMed, Scopus, Web of Science, ScienceDirect, Embase via Ovid, and Cochrane Library. The literature search was performed on August 13, 2025. The reviewer developed the search strategy under the guidance of a librarian. The search strategy combined Medical Subject Headings (MeSH) terms and free‐text words using the Boolean operators “AND” and “OR.” The search strategy was adapted for the other databases.

### 2.3 Screening and Study Selection

The initial search yielded a total of 1069 records. Duplicate records were removed. Two authors independently screened the titles and abstracts of the retrieved records using Rayyan [13]. Records were classified as included, excluded, or unclear. Full‐text screening was then performed for potentially eligible studies to confirm whether the inclusion criteria were met. During full‐text assessment, studies were excluded for reasons including protocol‐only reports, use of other hyaluronic acid fillers, or lack of clearly reported outcome data. Disagreements between reviewers were resolved through discussion, and unresolved conflicts were referred to the lead reviewer until consensus was reached. Eligible studies were subsequently forwarded to other authors for data extraction. The study selection process was presented using the PRISMA 2020 flow diagram.

### 2.4 Data Collection Process

Reviewers developed a structured and standardized data extraction form using Microsoft Excel to extract relevant information from the included studies following PRISMA guidelines. Three authors independently extracted information on: (1) study characteristics (country, study design, study period, population characteristics, sample size, study setting, follow‐up duration, number of visits, and optional retreatment [Yes/No]); (2) baseline characteristics of the target population (age, number of females, re‐touch treatment, and Fitzpatrick phototypes); (3) agent and technique (concentration and lidocaine content, injection technique(s), treatment area, total volume of initial and touch‐up treatments combined, total volume of initial treatment, and total volume of touch‐up treatment); (4) safety outcomes (overall adverse event rate, severity, disorders and administration site conditions, and relationship to the injection site); and (5) effectiveness outcomes (clinician‐assessed severity scales and patient‐reported outcomes). Additionally, the information regarding study funding sources, manufacturer involvement, and author conflicts of interest was extracted from the included studies whenever available. These data were summarized in a Supplementary File Table S2.

After data extraction, each of the three reviewers independently cross‐checked the extracted data. Conflicts were resolved through discussion among the reviewers. Finally, one reviewer reviewed the complete dataset to identify potential errors or inconsistencies and finalized the spreadsheet for statistical analysis.

### 2.5 Risk of Bias Assessment

To assess the risk of bias in the included studies, the Cochrane Risk of Bias 2 (RoB 2) tool was used for randomized controlled trials and the Cochrane Risk of Bias in Non‐Randomized Studies of Interventions (ROBINS‐I) tool was used for non‐randomized studies. Two independent reviewers assessed the risk of bias using the relevant tools. Any disagreements were discussed and resolved through consensus. The finalized assessments were documented, and relevant figures illustrating the risk of bias were generated.

### 2.6 Statistical Analysis

Meta‐analyses were performed using R software version 4.4.2. This study employed a single‐arm meta‐analysis to synthesize proportions and incidence rates derived from observed events and sample totals. The results were visualized using forest plots, which display the effect sizes of individual studies alongside the overall pooled estimate. This pooled estimate was calculated using a random‐effects model, with precision reflected by 95% confidence intervals (CIs). Continuous outcomes, including WSRS mean scores, were summarized as pooled mean estimates with 95% confidence intervals using random‐effects models. Heterogeneity was assessed using Cochran's Q test and the *I* ^2^ statistic, with *I* ^2^ values categorized as low (< 25%), moderate (25%–75%), or high (> 75%). Subgroup meta‐analysis was performed at multiple predefined follow‐up time points to evaluate changes in aesthetic outcomes over time.

## 3 Results

### 3.1 Search Results and Study Selection

A total of 1069 records were identified from these databases. After removing 496 duplicate records, 573 unique records remained for title and abstract screening. During this initial screening phase, we excluded 557 records as irrelevant to the review's objectives. We then retrieved the full texts of the remaining 16 articles and assessed them for eligibility. Upon reviewing the full texts, we excluded several studies for the following reasons: one study used others HY‐17.5 or other agents not under investigation; three involved mixed treatment arms that did not allow for clear data extraction; and one lacked clearly documented outcome data, among other exclusion criteria. Ultimately, 11 studies met all inclusion criteria and were included in the final systematic review. The study selection process is summarized in the PRISMA 2020 flow diagram (Figure 1).

**FIGURE 1:**
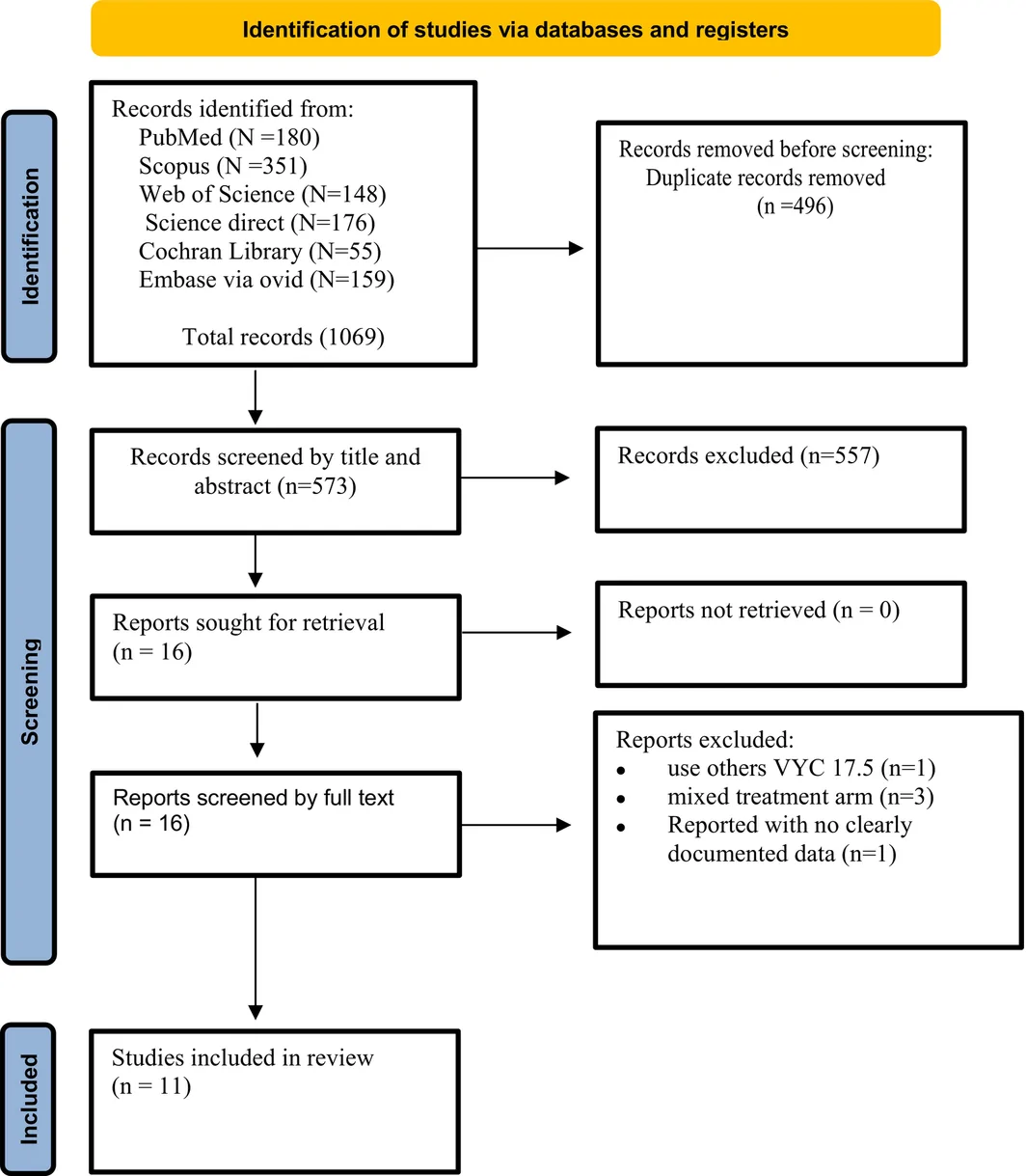
PRISMA chart representing the literature search and exclusion reports for study selection process.

### 3.2 Characteristics of Included Studies

Data from 11 included studies were analyzed, comprising a total of 967 participants. Individual study sample sizes ranged from 15 to 175 participants. Most participants were female, and the mean age across studies ranged from 42 to 59.2 years. The included studies evaluated VYC‐17.5L (Volift/Vollure) hyaluronic acid dermal filler across different facial aesthetic indications, with follow‐up assessments performed at baseline and multiple post‐treatment time points, ranging from early evaluations to long‐term follow‐up. Follow‐up completion rates were generally high across the included studies, with most studies reporting rates exceeding 90%.

The included studies reported adverse events using different assessment methods and follow‐up periods. Frequently reported events included edema, redness, pain, bruising, tenderness, itching, and nodules; however, their reported frequencies varied substantially across studies. Serious adverse events were rarely reported. The overall baseline characteristics are summarized in Table 1, and the characteristics of included studies are presented in Supplementary Table S1. Regarding the information of funding sources, manufacturer involvement, and reported conflicts of interest among the included studies was assessed and is summarized in Supplementary Table S2. Several included studies reported manufacturer sponsorship, product provision, or author relationships with the manufacturer, whereas other studies did not report relevant financial relationships.

**TABLE 1:** Bassline characteristic of included studies.

Study ID	Age		Number of females		Re‐touch					Fitzpatrick phototypes									
					Allowed (Y/N)	Type of HA	Time point (Day)	Population		I		II		III		IV		V	
	Mean	SD	Event	Total				Event	Total	Event	Total	Event	Total	Event	Total	Event	Total	Event	Total
Xie et al. 2022 [14]	42	7	163	171	Yes	Juvéderm Volift	0	63	171	—	—	—	—	—	—	—	—	—	—
Demosthenous et al. 2022 [15]	36.8	9.3	59	60	Yes	Juvéderm Volift	14	13	60	6	60	34	60	17	60	2	60	1	60
Del Cueto et al. 2025 [16]	55.4	10.7	73	80	No	Juvéderm Volift	—	—	—	1	80	33	80	28	80	18	80	—	—
Cueto et al. 2025 [17]	59.2	8.2	79	83	Yes	Juvéderm Volift	14	53	83	1	83	33	83	33	83	16	83	—	—
Dayan et al. 2019 [18]	54	8.33	117	123	No	Juvéderm Vollure	—	—	—	—	—	—	—	—	—	—		—	—
Rzany et al. 2019 [19]	57.9	8.12	75	90	Yes	Juvéderm Volift	14	8	30	0	0	35	90	50	90	5	90	—	—
Ogilvie et al. 2019 [20]	56	5.55	53	53	Yes	Juvéderm Volift	14	Rt: 49 Lt: 48	53	—	—	20	53	24	53	—	—	—	—
Monheit et al. 2018 [10]	56	12.5	117	123	Yes	Juvéderm Volift	30	63	123	14	123	27	123	31	123	20	123	18	123
Siperstein et al. 2024 [21]	42.3	9.48	11	15	Yes	Juvéderm Volift	30	15	15	—	—	—	—	—	—	—	—	—	—
Sattler et al. 2017 [22]	49.3	9.5	66	70	Yes	Juvéderm Volift	14	28	70	2	70	28	70	33	70	3	70	3	70
Alimohammadi et al. 2024 [23]	56.4	5.9	48	48	Yes	Juvéderm Volift	30	37	48	1	48	26	48	19	48	1	48	—	—

### 3.3 Agent and Technical Intervention for Studies

All included studies used VYC‐17.5L (Juvéderm Volift/Vollure), a hyaluronic acid‐based dermal filler formulated with a concentration of 17.5 mg/mL HA and 3 mg/mL lidocaine. The treatment sites varied across studies and included different facial regions for rejuvenation and contouring. The most frequently treated area was the nasolabial fold (NLF), followed by other facial areas including the lips, forehead, marionette lines, cheeks, and facial wrinkles.

All studies reported different injection techniques according to the treated anatomical region and the intended aesthetic outcome. These techniques included linear threading (antegrade and retrograde), fanning, serial puncture, and cross‐hatching. Linear threading and fanning were commonly used for nasolabial folds and lip augmentation, whereas fanning and bolus injection techniques were used for cheek augmentation.

The administered volume varied according to the anatomical site and treatment objective. The mean volume used for the initial nasolabial fold treatment ranged from 1.15 to 2.4 mL depending on the number of treated sites. For lip augmentation, the mean initial treatment volume was 1.6 mL, whereas cheek augmentation required a higher mean volume of 3.8 mL per cheek. Several studies allowed touch‐up treatments during follow‐up. The volume of touch‐up procedures was generally lower than the initial treatment, ranging from 0.265 to 5.2 mL for nasolabial folds and approximately 2.5 mL for per cheek, supporting maintenance of aesthetic outcomes after the initial treatment Table 2.

**TABLE 2:** Agent and technical intervention for studies.

Study ID	Agent/Filler/concentration	Injection technique(s)			Treatment area			Total volume of initial			Total volume of touch up		
		Technique	Event	Total	Area	Event	Total	Area	Mean	SD	Area	Mean	SD
Xie et al. 2022 [14]	VYC‐17.5L Juvéderm Volift (17.5 mg/mL + Lidocaine 3 mg/mL)	—	—	—	Nasolabial Folds	171	171	Nasolabial Folds	2.4	1.55	Nasolabial Folds	5.2	2.36
Demosthenous et al. 2022 [15].	VYC‐17.5L Juvéderm Volift (17.5 mg/mL + Lidocaine 3 mg/mL)	—	—	—	lip	51	51	lip	1.6	0.7	lip	1	0.2
Del Cueto et al. 2025 [16]	VYC‐17.5L Juvéderm Volift (17.5 mg/mL + Lidocaine 3 mg/mL)	Fanning and small bolus	80	80	forehead	75	80	—	—	—	—	—	—
Cueto et al. 2025 [17]	VYC‐17.5L Juvéderm Volift (17.5 mg/mL + Lidocaine 3 mg/mL)	Fanning and linear threading	83	83	Marionette line	83	83	—	—	—	—	—	—
Dayan et al. 2019 [18]	Juvéderm Vollure (17.5 mg/mL + Lidocaine 3 mg/mL)	Serial Puncture/Tunneling/Fanning/Cross—hatching.	114/62/39/20	123	Nasolabial Folds	123	123	Nasolabial Folds	1.4	0.48	Nasolabial Folds	0.7	0.225
Rzany et al. 2019 [19]	VYC‐17.5L Juvéderm Volift (17.5 mg/mL + Lidocaine 3 mg/mL)	—	90	90	Nasolabial folds	30	90	Nasolabial folds	1.19	Not reported			
Ogilvie et al. 2019 [20]	VYC‐17.5L Juvéderm Volift (17.5 mg/mL + Lidocaine 3 mg/mL)	Fanning	53	53	Rt, Lt cheek	53	53	Rt, Lt cheek	3.8	0.5	Rt, Lt cheek	2.5	0.75
Monheit et al. 2018 [10]	VYC‐17.5L Juvéderm Volift (17.5 mg/mL + Lidocaine 3 mg/mL)	Serial puncture/tunneling/fanning/cross hatching	114/62/39/20	123	Nasolabial folds	123	123	Nasolabial Folds	1.475	0.56	Nasolabial Folds	0.625	0.23
Siperstein et al. 2024 [21]	VYC‐17.5L Juvéderm Volift (17.5 mg/mL + Lidocaine 3 mg/mL)	Inject parallel in the dermis	15	15	Cheeks	15	15	—	—	—	—	—	—
Sattler et al. 2017 [22]	VYC‐17.5L Juvéderm Volift (17.5 mg/mL + Lidocaine 3 mg/mL)	Retrograde tunnel threading/Antegrade tunnel threading/Serial puncture/Fanning/Other	45/13/14/31/11	70	Nasolabial folds	70	70	Nasolabial Folds	2.4	0.5	Nasolabial Folds	1.6	0.5
Alimohammadi et al. 2024 [23]	VYC‐17.5L Juvéderm Volift (17.5 mg/mL + Lidocaine 3 mg/mL)	Linear threading	48	48	Nasolabial folds	48	48	Nasolabial Folds	1.15	0.32	Nasolabial Folds	0.88	0.2

### 3.4 Risk of Bias Assessment

The risk of bias assessment was performed using the Cochrane Risk of Bias 2 (RoB 2) tool for randomized controlled trials and the Cochrane Risk of Bias in Non‐Randomized Studies of Interventions (ROBINS‐I) tool for non‐randomized studies. Two independent reviewers assessed the risk of bias across the predefined domains of each tool. Any disagreements between reviewers were resolved through discussion and consensus.

Overall, the included studies demonstrated variability in the risk of bias across different domains. The majority of studies were considered to have a low or moderate risk of bias, while no studies were judged to have a critical risk of bias. For randomized controlled trials, the evaluated domains included bias arising from the randomization process, deviations from intended interventions, missing outcome data, measurement of outcomes, and selection of reported results. For non‐randomized studies, assessments included bias due to confounding, selection of participants, classification of interventions, deviations from intended interventions, missing data, outcome measurement, and selection of reported results.

The detailed risk of bias assessments, including ROBINS‐I summary plots and RoB 2 traffic light plots, are presented in the Supplementary Figures S1 and S2.

### 3.5 Clinician‐Assessed Aesthetic Outcomes

#### 3.5.1 GAIS (Global Aesthetic Improvement Scale) Subgroup Analysis by Follow‐Up Time

At five post‐treatment time points (1, 3, 6, 9, and 12 months), a subgroup analysis of clinician‐assessed Global Aesthetic Improvement Scale (GAIS) responder rates was performed according to follow‐up duration. The overall pooled analysis demonstrated significant heterogeneity among studies (*I* ^2^ = 94.7%, *p* < 0.0001). A significant difference was observed between follow‐up time points, as indicated by the test for subgroup differences (*χ* ^2^ = 156.63, df = 4, *p* < 0.0001).

At 1 month (4 studies), responder rates ranged from 71% (95% CI: 56%–83%) to 99% (95% CI: 93%–100%), with high heterogeneity (*I* ^2^ = 89.5%, *p* < 0.0001). At 3 months (4 studies), heterogeneity was low (*I* ^2^ = 0%, *p* = 0.92), and responder rates ranged from 87% (95% CI: 75%–94%) to 90% (95% CI: 77%–97%). At 6 months (5 studies), responder rates ranged from 70% (95% CI: 59%–80%) to 94% (95% CI: 84%–99%), with moderate‐to‐high heterogeneity (*I* ^2^ = 72.1%, *p* = 0.008). At 9 months (4 studies), responder rates ranged from 39% (95% CI: 28%–50%) to 73% (95% CI: 58%–85%), with high heterogeneity (*I* ^2^ = 84.1%, *p* = 0.0003). At 12 months (2 studies), responder rates were 33% (95% CI: 17%–53%) and 24% (95% CI: 15%–35%), with low heterogeneity (*I* ^2^ = 3.6%, *p* = 0.31).

Overall, clinician‐assessed GAIS responder rates demonstrated a progressive decline over follow‐up time, with the highest pooled responder rates and lowest heterogeneity observed at 3 months after treatment. Figure 2.

**FIGURE 2:**
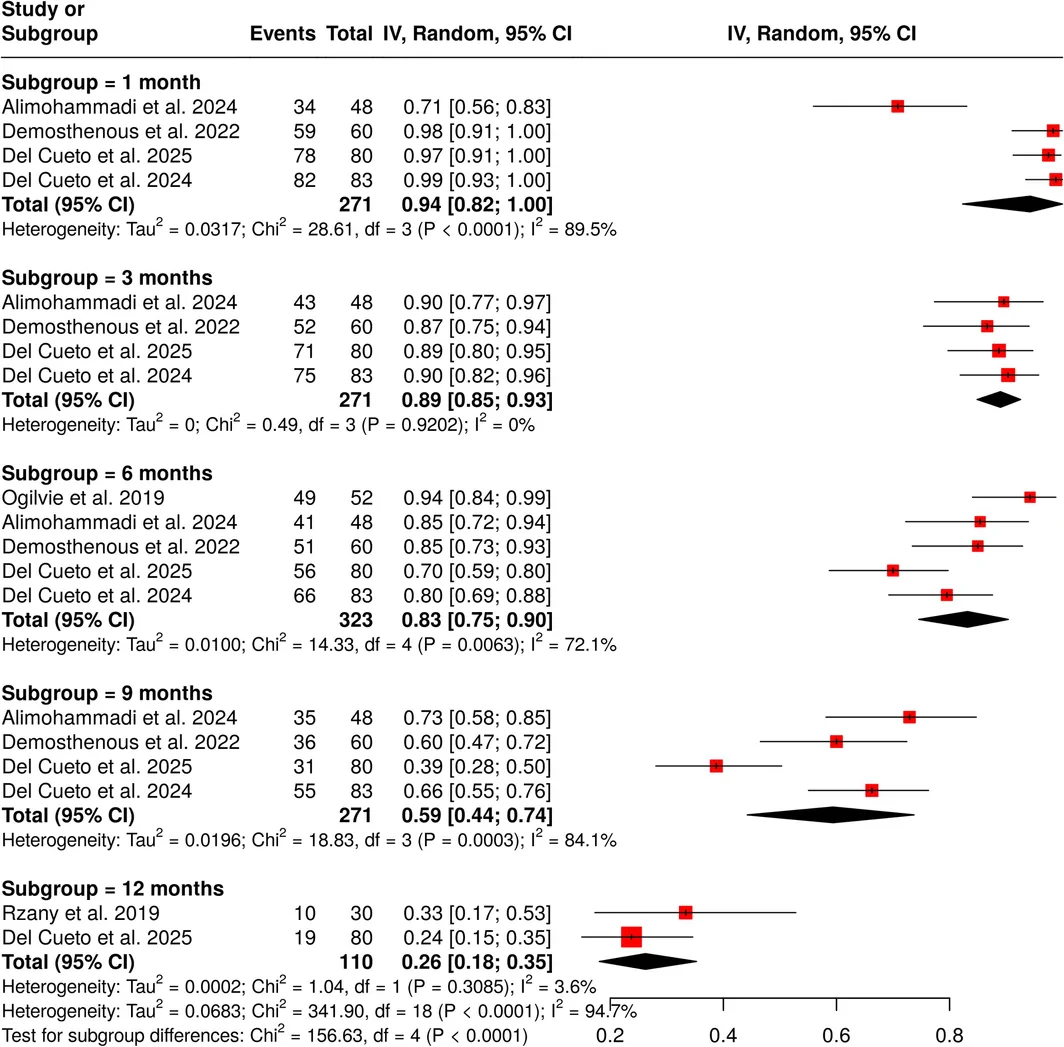
Forest plot of the Global Aesthetic Improvement Scale (GAIS) subgroup analysis by follow‐up time.

#### 3.5.2 Nasolabial Fold Severity Scale(NLFSS)

Responder rates based on the NLFSS for nasolabial fold correction were evaluated at 1, 3, and 6 months after treatment. The overall pooled analysis showed moderate heterogeneity across all follow‐up periods (*I* ^2^ = 67.7%, *p* = 0.009). Subgroup analysis according to follow‐up duration demonstrated no significant difference between time points (*χ* ^2^ = 1.95, df = 2, *p* = 0.38).

At 1 month (2 studies), responder rates were high, reaching 98% (95% CI: 94%–100%) in Monheit et al. and 91% (95% CI: 85%–95%) in Dayan et al. The pooled estimate was 95% (95% CI: 86%–100%), with significant heterogeneity (*I* ^2^ = 85.6%, *p* = 0.008). However, at 3 months (2 studies), responder rates remained consistently high, with 91% (95% CI: 85%–95%) in Monheit et al. and 89% (95% CI: 83%–94%) in Dayan et al. The pooled estimate was 90% (95% CI: 86%–94%), without evidence of heterogeneity (*I* ^2^ = 0%, *p* = 0.67).

At 6 months (2 studies), both studies reported responder rates of 89% (95% CI: 82%–94%). The pooled estimate was 89% (95% CI: 84%–92%), with no heterogeneity detected (*I* ^2^ = 0%, *p* = 1.00).

Overall, NLFSS responder rates remained high and stable (≥ 89%) throughout the 6‐month follow‐up period, indicating sustained improvement in nasolabial fold severity after VYC‐17.5L treatment. Figure 3.

**FIGURE 3:**
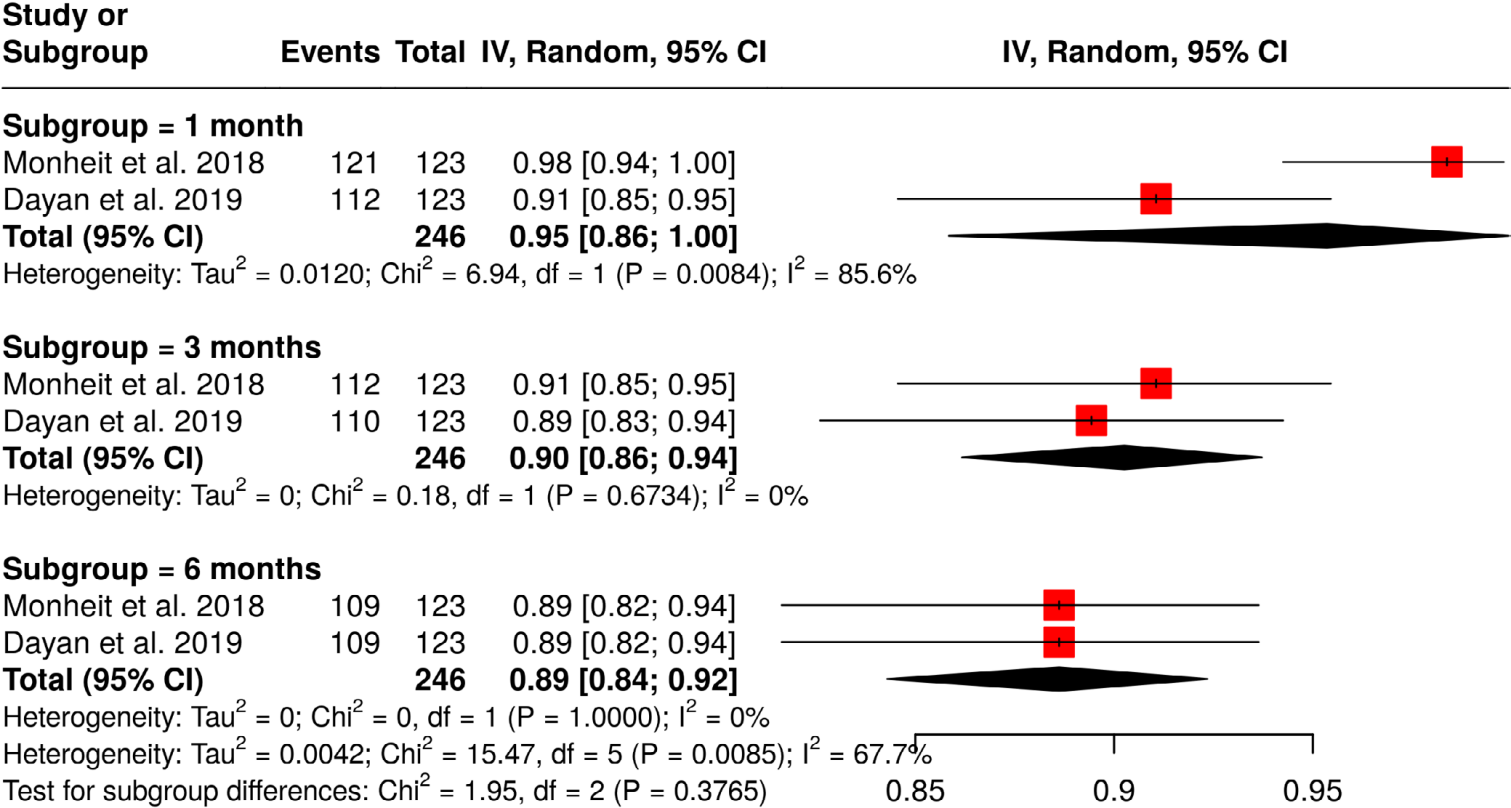
Forest plot of the Nasolabial Fold Severity Scale (NLFSS).

### 3.6 Patient‐Reported Outcomes

#### 3.6.1 Global Aesthetic Improvement Scale (GAIS) Subgroup Analysis by Follow‐Up Time

Patient‐reported GAIS responder rates were assessed at 1, 3, 6, 9 and 12 months' post‐treatment. The overall pooled analysis was highly heterogeneous (*I* ^2^ = 91.4%, *p* < 0.0001). Subgroup analysis by duration of follow‐up was highly significant (*χ* ^2^ = 75.60, df = 4, *p* < 0.0001), confirming the strong influence of timing of assessment on patient‐reported outcomes. 1 month (3 studies): Response rate ranged from 88 (95% CI: 78–94) to 98 (95% CI: 91–100); pooled 95 (95% CI: 87–99); *I* ^2^ = 72.8%, *p* = 0.03, moderate‐to‐high heterogeneity. 3 months (3 studies): Response rate 82%–87%; pooled 85 (95% CI: 79%–90%); *I* ^2^ = 0%, *p* = 0.79, no heterogeneity. 6 months (4 studies): Response rate 75%–98%; pooled 86 (95% CI: 74%–95%); *I* ^2^ = 82.4%, *p* = 0.0007, high heterogeneity. Nine months (4 studies): Response rate 51%–69%; pooled 62 (95% CI: 53%–71%); *I* ^2^ = 53.6%, *p* = 0.09, moderate heterogeneity. 12 months (2 studies): Response rates were 51% and 54%; pooled 53 (95% CI: 45–60), *I* ^2^ = 0%, *p* = 0.75, no heterogeneity. In summary, patient‐reported aesthetic improvement remained high and relatively stable through 6 months (pooled responder rates 85%–95%), followed by a substantial decline to approximately 50%–60% at 9 and 12 months Figure 4.

**FIGURE 4:**
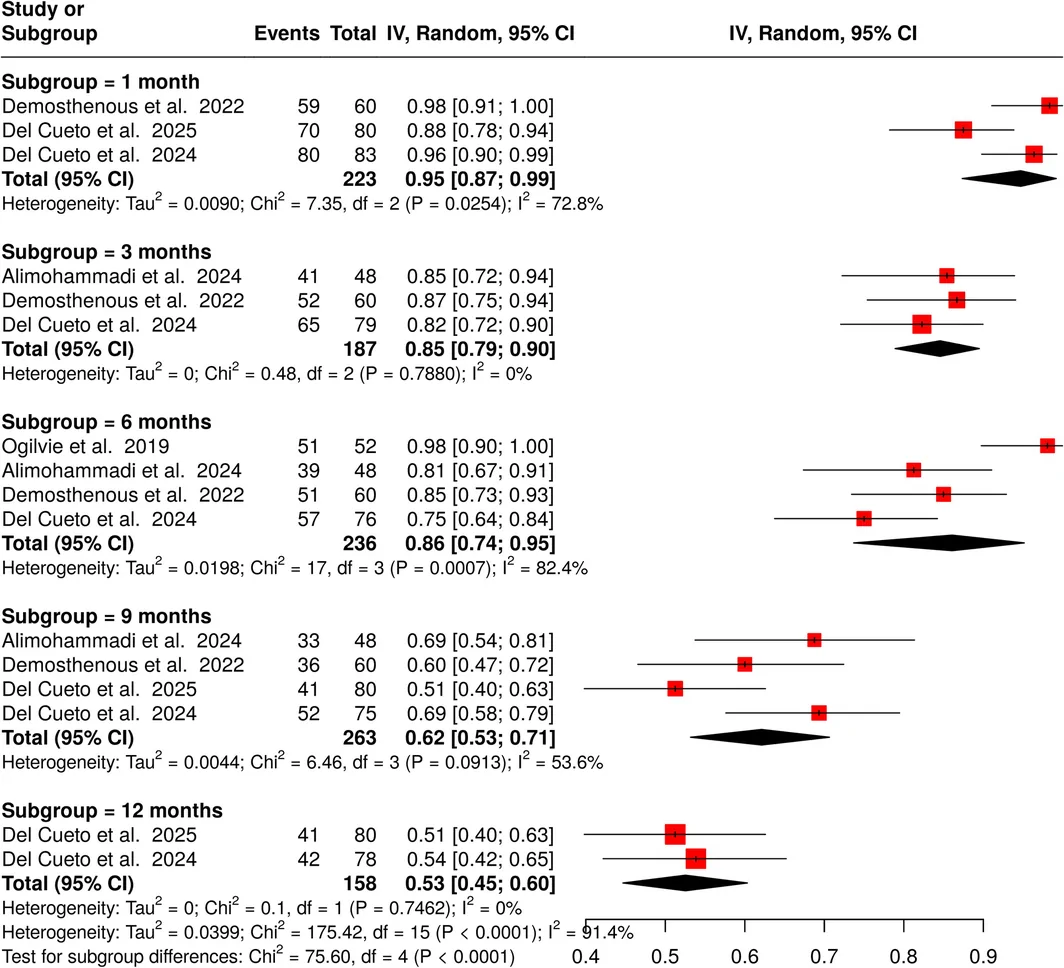
Forest plot of patient‐reported GAIS responder rates subgroup analysis by follow‐up time.

#### 3.6.2 FACE‐Q Subgroup Analysis by Follow‐Up Time

A subgroup analysis of patient‐reported outcomes was performed using the FACE‐Q scale across five post‐treatment time points: 1, 3, 6, 9, and 12 months. The pooled analysis across all studies and follow‐up periods demonstrated moderate heterogeneity (*I* ^2^ = 63.7%, *p* = 0.0001). Subgroup analysis by follow‐up duration showed a significant difference between time points (*χ* ^2^ = 31.28, df = 4, *p* < 0.0001), indicating differences in responder rates across follow‐up periods.

At 1 month (3 studies), responder rates were consistently high, ranging from 98% (95% CI: 91%–100%) to 100% (95% CI: 95%–100%), with no heterogeneity (*I* ^2^ = 0%, *p* = 0.41). At 3 months (5 studies), responder rates ranged from 87% (95% CI: 75%–94%) to 96% (95% CI: 91%–99%), with low‐to‐moderate heterogeneity (*I* ^2^ = 36.4%, *p* = 0.18). At 6 months (5 studies), responder rates ranged from 85% (95% CI: 73%–93%) to 95% (95% CI: 90%–98%), with low‐to‐moderate heterogeneity (*I* ^2^ = 37.1%, *p* = 0.17). At 9 months (2 studies), both studies reported responder rates of 90% (95% CI: 81%–96% and 82%–96%, respectively), with no heterogeneity (*I* ^2^ = 0%, *p* = 0.94). At 12 months (3 studies), responder rates remained high, ranging from 91% (95% CI: 85%–95%) to 94% (95% CI: 86%–98%), with no heterogeneity (*I* ^2^ = 0%, *p* = 0.72).

Compared with clinician‐assessed GAIS outcomes, patient‐reported satisfaction measured by FACE‐Q remained consistently high throughout the 12‐month follow‐up period. The lowest point estimate reported across all study time points was 85% at 6 months, indicating sustained patient‐reported satisfaction following treatment: Figure 5.

**FIGURE 5:**
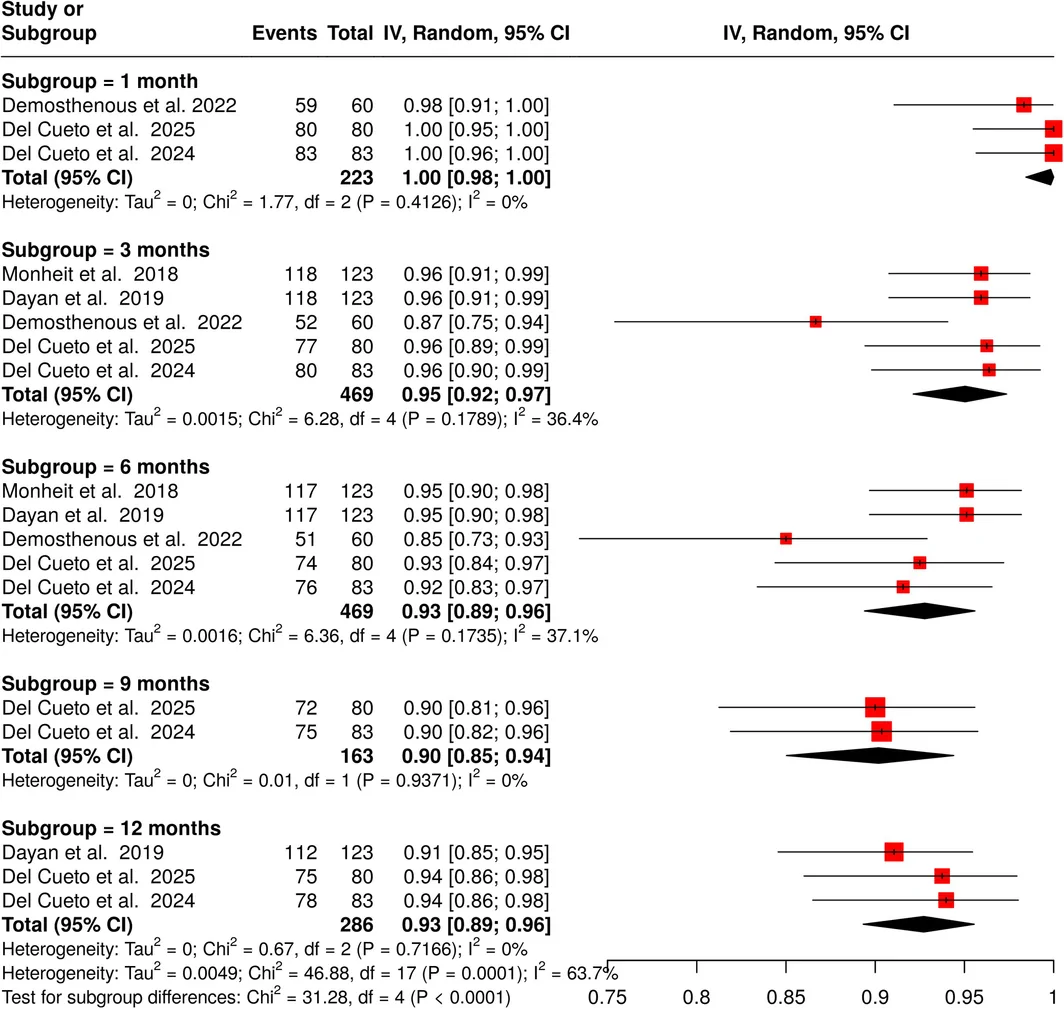
Forest plot of the FACE‐Q with subgroup analysis by follow‐up time.

#### 3.6.3 Patient Satisfaction Scales (NLFs) Subgroup Analysis by Follow‐Up Time

Patient‐reported satisfaction for the NLF area was reported at 1, 3, 6, and 9 months after treatment. The overall pooled analysis showed heterogeneity across studies (*I* ^2^ = 84.6, *p* = 0.0001). Subgroup analysis by time point did not show a statistically significant difference over time (χ^2^ = 5.43, df = 3, *p* = 0.1431).

At 1 month (2 studies), satisfaction rates were highly variable, with 93% (95% CI: 87%–97%) reported in Monheit et al. (2018) and 52% (95% CI: 37%–67%) in Alimohammadi et al. (2024). The pooled estimate was 76% (95% CI: 29–100), with significant heterogeneity (*I* ^2^ = 96.9%, *p* = 0.0001).

At 3 months (2 studies), patient satisfaction remained high across the included studies, with 81% reported in Monheit et al. (2018) and 75% in Alimohammadi et al. (2024). The pooled estimate was 80% (95% CI: 0.73–0.85), with no heterogeneity (*I* ^2^ = 0%, *p* = 0.3511).

At 6 months (3 studies), satisfaction remained stable, with rates of 78% in Monheit et al. (2018), 69% in Alimohammadi et al. (2024), and 78% in Dayan et al. (2019). The pooled estimate was 77% (95% CI: 72%–81%), with no heterogeneity detected (*I* ^2^ = 0%, *p* = 0.40).

Overall, patient satisfaction with nasolabial fold treatment remained high during the available follow‐up periods, particularly at 3 and 6 months. However, interpretation of later follow‐up outcomes was limited by the small number of available studies: Figure 6.

**FIGURE 6:**
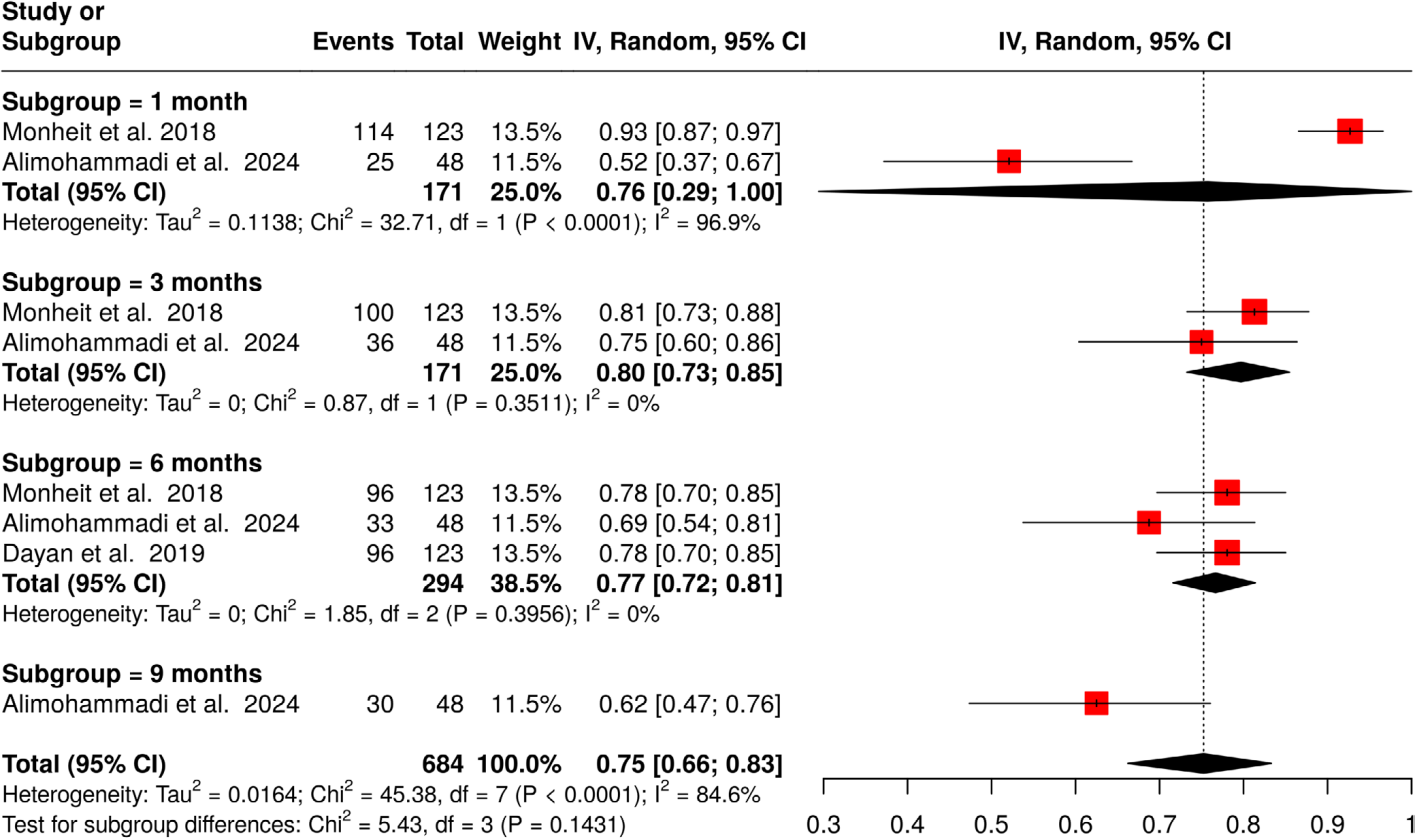
Forest plot of the Patient Satisfaction Scales to (NLFs) subgroup analysis by follow‐up time.

#### 3.6.4 Responder Rate (≥ 1‐Point Improvement) Subgroup Analysis by Follow‐Up Time

Responder rates were evaluated at 1, 3, 6, 9, and 12 months after treatment. Overall, the pooled analysis showed significant heterogeneity (*I* ^2^ = 91.4%, *p* < 0.0001). Subgroup analysis by time point was statistically significant (χ^2^ = 18.09, df = 4, *p* = 0.001), confirming that follow‐up duration was a significant source of heterogeneity. At 1 month (4 studies), responder rates ranged from 70% to 99%, with a pooled responder rate of 94% (95% CI: 80%–100%) and high heterogeneity (*I* ^2^ = 94.0%, *p* < 0.0001). At 3 months (3 studies), responder rates ranged from 68% to 94%, with a pooled responder rate of 85% (95% CI: 68%–96%) and high heterogeneity (*I* ^2^ = 88.2%, *p* = 0.0002). At 6 months (3 studies), responder rates ranged from 72% to 85%, with a pooled responder rate of 82% (95% CI: 76%–88%) and low heterogeneity (*I* ^2^ = 32.6%, *p* = 0.23). At 9 months (2 studies), responder rates were 65% and 84%, with a pooled responder rate of 75% (95% CI: 55%–91%) and moderate‐to‐high heterogeneity (*I* ^2^ = 78.3%, *p* = 0.03). At 12 months (2 studies), responder rates were 64% and 65%, with a pooled responder rate of 65% (95% CI: 56%–73%) and no heterogeneity (*I* ^2^ = 0%, *p* = 0.93). Responder rates were highest shortly after treatment (pooled responder rate of 94% at 1 month) and gradually decreased over time to 65% at 12 months. The greatest decline occurred between 1 and 3 months, followed by a slower decline thereafter Figure 7.

**FIGURE 7:**
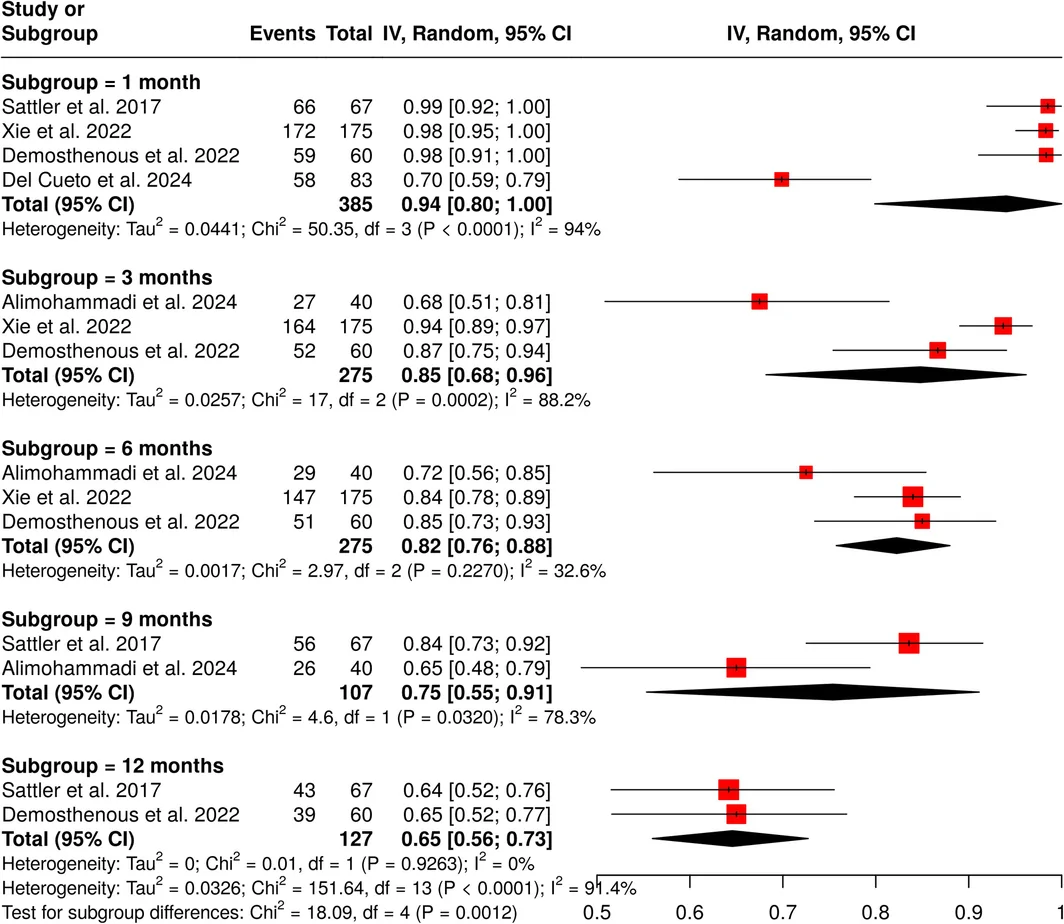
Forest plot of the Responder rate (≥ 1‐point improvement) subgroup analysis by follow‐up time.

#### 3.6.5 Wrinkle Severity Rating Scale(WSRS) Subgroup Analysis by Follow‐Up Time

WSRS mean scores were compared at 6 and 9 months after treatment. The overall pooled comparison indicated substantial between‐study heterogeneity (*I* ^2^ = 98.6%, *p* < 0.0001). Subgroup analysis indicated no statistically significant difference between time points (χ^2^ = 0.01, df = 1, *p* = 0.92). At 6 months (2 studies), Alimohammadi et al. (2024) reported a mean score of 2.75 (95% CI: 2.55–2.95), whereas Rzany et al. (2019) reported a mean score of 1.60 (95% CI: 1.56–1.64). The pooled mean was 2.17 (95% CI: 1.04–3.30), with high heterogeneity (*I* ^2^ = 99.2%, *p* < 0.0001). At 9 months (2 studies), Alimohammadi et al. (2024) reported a mean score of 2.70 (95% CI: 2.47–2.93), whereas Rzany et al. (2019) reported a mean score of 1.80 (95% CI: 1.73–1.87). The pooled mean was 2.24 (95% CI: 1.36–3.13), with high heterogeneity (*I* ^2^ = 98.1%, *p* < 0.0001). Within each study, WSRS mean scores showed minimal change between 6 and 9 months. However, a consistent difference in scores was observed between studies at each time point, with Rzany et al. reporting WSRS scores approximately 1 point lower than Alimohammadi et al. This difference between‐study variability resulted in wide pooled confidence intervals and limited interpretation of the overall mean comparison Figure 8.

**FIGURE 8:**
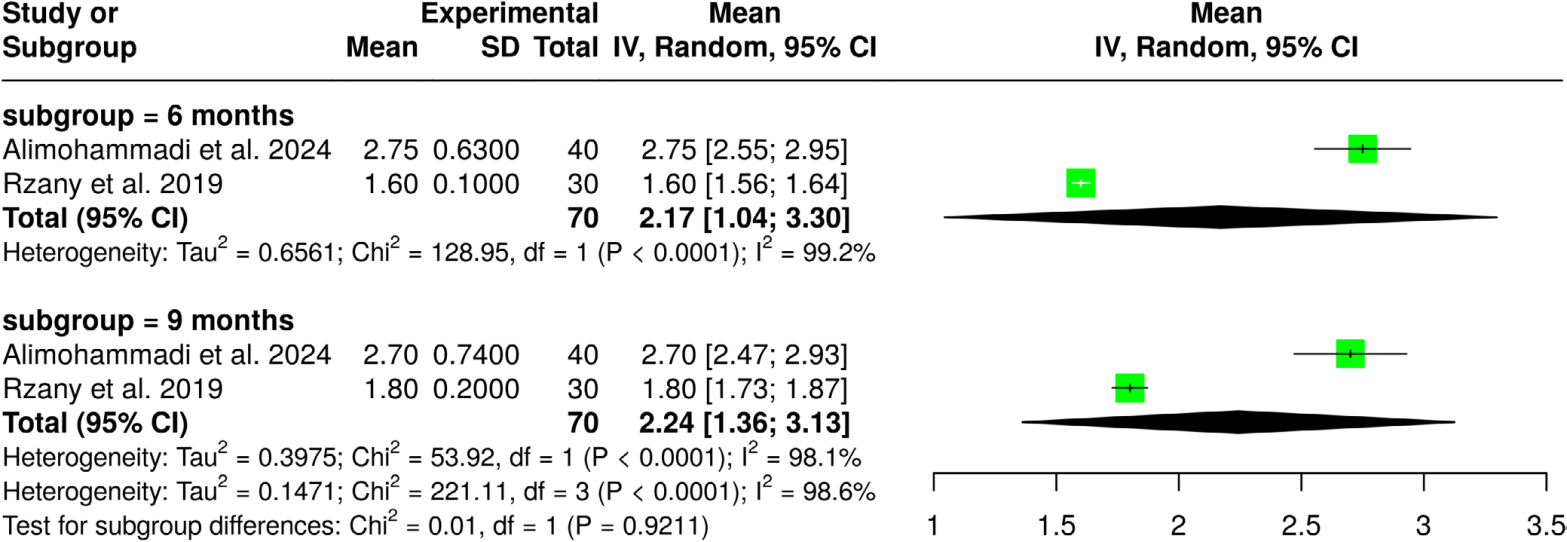
Forest plot of the Wrinkle Severity Rating Scale (WSRS).

### 3.7 Safety Outcomes

#### 3.7.1 Adverse Events

Adverse events following VYC‐17.5L treatment were generally mild and related to the injection procedure. Local reactions, including bruising, pain, swelling, redness, tenderness, itching, and lumps, were among the most frequently reported events. Substantial differences were observed between patient‐reported and investigator‐reported incidences across adverse event categories. Patient‐reported rates were consistently higher than investigator‐assessed rates, resulting in considerable heterogeneity and limiting the clinical interpretation of overall pooled estimates. These findings highlight the important influence of the assessment method on the reported incidence of local adverse events. Detailed analyses of individual adverse events and corresponding forest plots are provided in the Supplementary Materials.

## 4 Discussion

Our study comprehensively evaluated the efficacy and safety of HA (Volift/Vollure) for Facial Enhancement. Our results, using multiple clinician‐ and patient‐reported outcomes, revealed several distinct outcomes.

The findings identified some significant trends in the effectiveness of the hyaluronic acid treatment. The observed peak of clinician‐assessed GAIS at early post‐treatment time points likely reflects the immediate volumizing effect of the filler, which may reflect the gradual degradation of filler material and changes in tissue response over time. In contrast, patient‐reported outcomes, including GAIS and FACE‐Q satisfaction, remained high for up to 6 months. The lingering elevated improvement in NLFSS suggests that nasolabial folds respond particularly well and predictably to treatment, while the relative stability in WSRS scores between 6 and 9 months reflects beneficial effects on broader wrinkle severity despite interstudy differences. The decline in response rates over follow‐up suggests that most of the aesthetic benefit accrued in the early work, making early follow‐up and reinjections critical.

A previous meta‐analysis by Stefura et al. concerning safety and efficacy of HA for nasolabial folds reported improvement in the Global Aesthetic Improvement Scale (GAIS), with peak aesthetic outcomes observed within the first 3–5 months post‐treatment [24]. In comparison, the current study also demonstrated a time‐dependent decline in clinician‐assessed GAIS, with responder rates peaking at 87%–90% at 3 months and declining sharply to 24%–33% at 12 months, suggesting a more pronounced reduction over time. Our study provides more robust evidence by reporting direct and clear NLFSS values (≥ 89% improvement through 6 months), whereas the previous meta‐analysis inferred NLFSS improvement indirectly via WSRS [24]. Both studies indicated relative stability in WSRS between 6–9 months, despite substantial inter‐study variability, suggesting persistence of objective improvement in nasolabial fold correction even as subjective aesthetic ratings declined over time.

In a comparable manner, a randomized clinical trial (RCT) by Sundaram et al. reported persistently high patient satisfaction with FACE‐Q scores above 87% at one year [25]. The current study similarly demonstrated high satisfaction during the first 6 months (85%–100% for FACE‐Q, 85%–95% for GAIS), but a notable decline was observed at 9–12 months (~53%–62%). Nasolabial fold‐specific satisfaction remained consistent in the present study at 3–6 months (~77%–80%) but showed variability at other assessment points, whereas Sundaram et al. observed sustained improvement throughout the follow‐up period [25]. These variations may be attributed to differences in filler composition, injection methodology, study protocols, or patient demographics, highlighting the necessity for standardized outcome measurements and extended follow‐up in aesthetic medicine research.

In earlier study by Peng et al. patient‐reported outcomes were evaluated at week 24 following touch‐up injections, revealing no significant difference in GAIS 1‐point improvement between monophasic and biphasic hyaluronic acid formulations, though overall patient satisfaction was significantly higher with monophasic HA [26]. By contrast, the current study evaluated patient‐reported outcomes across multiple time points (1, 3, 6, 9, and 12 months) and demonstrated elevated satisfaction and GAIS improvement during the first 6 months (85%–100% for FACE‐Q, 85%–95% for GAIS), followed by a marked decline at 9–12 months (~53%–62%). Furthermore, nasolabial fold‐specific satisfaction in the present study was stable at 3–6 months (~77%–80%) but demonstrated considerable variability at other time points, while Peng et al. reported only general satisfaction at a single time point without regional differentiation [26]. These differences emphasize the significance of follow‐up duration, temporal evaluation, and region‐specific outcome measurements in assessing patient perception, suggesting that long‐term patient‐reported satisfaction may fluctuate based on study design, HA formulation, and assessment methodology.

In alignment with these findings, a study by Kaminer et al. in which patient‐reported GAIS responder rates (≥ 1 point improvement) were 95%–98% at 6 weeks and decreased to 72%–92% at 12 months, demonstrating that most patients maintained meaningful aesthetic improvement over 1 year [27]. In the current study, Responder Rate (≥ 1 point improvement) was 94% at 1 month post‐treatment and gradually declined to 65% at 12 months, with the most pronounced decrease occurring between 1 and 3 months. While both studies demonstrate an early peak in improvement followed by a gradual decline, the current study shows a slightly lower Responder Rate at 12 months, potentially reflecting variations in patient population, filler type, or assessment methodology. These findings collectively reinforce the consistent pattern of early high response with progressive reduction over time in patient‐perceived aesthetic outcomes.

### 4.1 Safety Profile

Adverse events following treatment were predominantly mild and temporary in nature, including bruising, redness, swelling, pain, tenderness, lumps, itching, induration, and discoloration. The reported incidence varied based on the evaluator: clinician‐reported rates remained low (0%–7%), while patient‐reported rates were considerably higher (20%–88%), with substantial variation across different studies. The observed discrepancy between patient‐reported and investigator‐reported adverse events should be interpreted cautiously, as these assessments capture different dimensions of the post‐treatment experience. Patient‐reported outcomes in the included studies were often collected through structured diaries or questionnaires, allowing the detection of transient symptoms and subjective sensations that may not be captured during scheduled clinical assessments. In contrast, investigator‐reported adverse events generally relied on predefined clinical criteria, severity thresholds, or the need for clinical intervention. Therefore, differences in assessment methodology, symptom duration, severity thresholds, and patient expectations may contribute to the observed variation between reporting methods. Accordingly, patient‐reported outcomes should not be interpreted as an overestimation of adverse events, but rather as complementary information reflecting the patient's experience and symptom burden following treatment. In a previous systematic review and meta‐analysis of randomized controlled trials evaluating hyaluronic acid lip fillers, it was found that approximately 50% of patients experienced adverse events, with swelling being most prevalent (78%), followed by firmness (48%), bruising (34%), and tenderness (33%), while serious reactions were rare (~1.5%) [28]. In the current study, adverse events were similarly reported, with clinician‐assessed frequencies consistently low (0%–7%) and patient‐reported frequencies markedly higher (20%–88%), including bruising, redness, swelling, pain, tenderness, lumps, itching, induration, and discoloration. Both studies emphasize that adverse events are typically mild and transient; however, the disparity between clinician‐ and patient‐reported rates in the current study emphasizes the importance of incorporating patient‐reported outcomes for a comprehensive safety evaluation.

The interpretation of the safety profile of VYC‐17.5L should also consider the specific physicochemical characteristics of Vycross technology. Unlike earlier hyaluronic acid fillers composed predominantly of high‐molecular‐weight HA, Vycross products combine low‐ and high‐molecular‐weight HA fractions through crosslinking technology, which contributes to their rheological properties and tissue integration. However, low‐molecular‐weight HA fragments have been described in immunological literature as biologically active molecules that may participate in innate immune signaling pathways [29, 30, 31, 32, 33].

Independent clinical literature has reported delayed inflammatory reactions, including delayed‐onset nodules and edema, following Vycross‐based filler injections, including reports involving VYC‐17.5L. These events may occur beyond the conventional follow‐up periods used in many clinical trials and therefore may not be fully captured by short‐term safety assessments [34, 35, 36, 37]. Accordingly, the favorable safety profile observed in this meta‐analysis should be interpreted within the context of the available observation periods and should not be extrapolated as evidence of long‐term inflammatory safety. Given that several included studies involved manufacturer sponsorship or reported professional relationships with the manufacturer, these factors should be considered when interpreting the certainty and generalizability of the available evidence. However, industry involvement alone does not necessarily indicate biased results and should be interpreted within the broader context of study design, methodology, and consistency of findings.

### 4.2 Limitations

This systematic review with meta‐analysis has several limitations. Substantial heterogeneity was observed among most outcomes, particularly in clinician‐ and patient‐reported measures, which may affect the interpretation and reliability of pooled estimates. The observed differences between clinician assessments and patient‐reported outcomes, especially regarding adverse events such as swelling, pain, and bruising, highlight the influence of assessment methodology on reported findings. The included studies varied in design, sample size, injection techniques, follow‐up duration, treatment areas, and outcome measurement tools, which limited comparability across studies and restricted the evaluation of long‐term effectiveness. Furthermore, some studies included relatively small participant groups, and adverse event reporting was inconsistent, reducing the interpretability of pooled safety outcomes. The limited number of studies available for some outcomes also restricted further statistical analyses, such as assessment of publication bias. Additionally, because this review focused on a specific commercially available product, the possibility of selective publication of favorable efficacy or safety outcomes cannot be excluded, particularly when considering the involvement of industry sponsorship in some included studies. Another limitation Some outcomes could not be quantitatively synthesized due to the limited number of available studies. Finally, restricting the review to English‐language publications and studies reporting separate VYC‐17.5L outcomes may have introduced potential selection bias.

### 4.3 Conclusion

VYC‐17.5L demonstrates a positive favorable safety profile and delivers aesthetic advantages for facial rejuvenation procedures. While patient satisfaction remains high during the early and intermediate follow‐up periods, objective aesthetic improvements demonstrate a progressive decline over time, underscoring the temporary nature of filler effects. These findings support VYC‐17.5L as an effective option for minimally invasive facial enhancement while emphasizing the importance of patient counseling regarding the expected duration and variability of treatment outcomes.

## Author Contributions

All authors contributed to the study conception and design. The idea was identified by A.A. Shimal. Validation of the idea was performed by M. Aljameie and K.M. Al‐Dhayani. S.M. Altbali was the leader of the team. The search strategy and database access were performed by E. Aljubori. Screening of titles, abstracts, and full texts was performed by S.M. Altbali and M. Hamdy. Excel sheet preparation and data collection were performed by A.M. Oudah. Data collection and risk of bias assessment were performed by M.A. Hussein and J. Firdous. Analysis was performed by Y. Alswaiti. Tables were prepared by M. Qaq. The manuscript was written by three authors: the introduction by S.M.M. Abdelbar, the results by M.S. ur Rehman, and the methods, discussion, and abstract by I. Yassin. Final review of the manuscript was performed by S.M. Altbali. All authors approved the final version for submission.

## Funding

The authors have nothing to report.

## Ethics Statement

The authors have nothing to report.

## Consent

The authors have nothing to report.

## Conflicts of Interest

The authors declare no conflicts of interest.

## Supporting information


**Table S1:** Summary of Characteristics of the included studies.
**Table S2:** Industry Sponsorship, Manufacturer Involvement, and Conflict‐of‐Interest Assessment of the Included Studies.
**Figure S1:** Risk of bias assessment by ROBINS‐I. (A) distribution for individual studies, (B) the summary of assessment.
**Figure S2:** Risk of bias assessment by RoB2. (A) distribution for individual studies, (B) the summary of assessment.
**Figure S3:** Forest plot of the bruising.
**Figure S4:** Forest plot of the discoloration.
**Figure S5:** Forest plot of the induration.
**Figure S6:** Forest plot of the itching.
**Figure S7:** Forest plot of the lumps sensitivity analysis.
**Figure S8:** Forest plot of the Pain after injection sensitivity analysis.
**Figure S9:** Forest plot of the redness sensitivity.
**Figure S10:** Forest plot of the swelling sensitivity analysis.
**Figure S11:** Forest plot of the Tenderness to touch sensitivity analysis.

## Data Availability

The data that support the findings of this study are available from the corresponding author upon reasonable request.
