# Supplementary material for: Efficacy, Durability, and Safety of VYC‐17.5L Hyaluronic Acid Dermal Filler for Facial Enhancement: A Systematic Review and Single‐Arm Meta‐Analysis
Source: J Cosmet Dermatol. 2026 Oct 11;25(10):e71233. doi: 10.1111/jocd.71233 (PMC13655297; doi:10.1111/jocd.71233)
Supplement: Supplementary file 1 [file JOCD-25-e71233-s001.docx]

**Table S1.** summary of Characteristics of the included studies.

| **Study ID** | **Country/**  **Study Design/**  **Study Period** | **Sample Size** | **Study Setting** | **Follow-up Duration** | **No. of Visits** | **Optional Re-treatment (Yes/No)** | **Outcome Measures** | **Inclusion Criteria** | **Exclusion Criteria** |
| --- | --- | --- | --- | --- | --- | --- | --- | --- | --- |
| **Xie et al., 2022** | China.  Prospective multicenter randomized controlled double-blind.  January 2016 – June 2017. | 175 | Hospital | at 12 months | 8 | No | NLFs, NFSS, GAIS | Age ≥18 years, with nasolabial folds, NLF severity improvement ≥1-point, NLFSS score | prior treatments included temporary dermal fillers, botulinum toxin injections, semipermanent or permanent facial implants, mesotherapy, and previous cosmetic procedures involving the face or neck. |
| **Demosthenous et al. 2022** | Portugal and United Kingdom.  Prospective, open-label, multicenter.  December 2018 – June 2020. | 60 | Private Dermatology Clinic | at 1, 3, 6, 12 months | 6 | Yes | LFS2, FACE-Q, GAIS | Aged ≥18 years, 1-point in LFS2, baseline grade minimal–mild moderate | pregnant or nursing, or with history of oral or facial plastic surgery, facial implants, dermal fillers, mesotherapy, cosmetic resurfacing, botulinum toxin injections below the orbital rim, or anticoagulant therapy. |
| **Del Cueto et al., 2025** | Lyon, France.  Prospective, open-label, single-group, post-marketing study.  September 2021 – November 2022. | 80 | Private Dermatology Clinic | at 1, 3, 6, 9, 12 months | 7 | Yes | GAIS, FACE-Q score | Adults ≥18, forehead irregularities, no other facial procedures | Pregnant/nursing, recent facial surgery or implants, cosmetic resurfacing, medication use, or active infection/inflammation (acne, herpes, cancerous or precancerous lesion) |
| **Del Cueto et al., 2024** | Lyon, France.  Prospective, open-label, single-group, post-marketing study.  September 2021 – November 2022. | 130 | Private Dermatology Clinic | at 1, 3, 6, 9, 12 months | 7 | Yes | AMLS, FACE-Q, GAIS | Adults ≥18 years, mild-to-very severe marionette lines no other procedures/treatments, negative pregnancy test, affiliation with health social system | undergone facial plastic surgery or received permanent facial, undergone cosmetic resurfacing (i.e., laser, photomodulation, intense pulsed light radiofrequency, dermabrasion, and chemical peel)) |
| **Dayan et al. 2019** | United States.  Prospective, multicenter, randomized, within-patient controlled study.  October 2013 – October 2015. | 123 | Private Dermatology Clinic | Up to 20 months | 11 | Yes | NLFSS, FACE-Q, Patient Satisfaction, ISRs, AEs | Adults ≥18 years, moderate/severe NLFs (score 2–3 on 5-point Allergan NLF Severity Scale) | previously facial surgery , undergone cosmetic resurfacing, pre-treatment with dermal fillers |
| **Rzany et al. 2019** | France.  Prospective, monocentric, split-face, double-blind, randomized controlled trial.  February2014 – September 2015. | 90 | Research organization specialized in dermatological clinical studies | at 18 months | 9 | Yes | WSRS, GAIS, FACE-Q | Patients with moderate to severe bilateral nasolabial folds (WSRS score 3 or 4) | Diabetic, autoimmune disease, filler allergy, coagulation disorder, recent anti-inflammatory or anticoagulant use, facial structural alterations, permanent or recent fillers, botulinum toxin, peeling, laser, or ultrasound treatment, or participation in another NLF trial. |
| **Ogilvie et al. 2019** | France.  Prospective open label study.  Oct 13 to Dec 14,2016. | 53 | Private Dermatology Clinic | at 45 days | 3 | Yes | GAIS - SPA(self-perception of age) | women aged 40–65 years with radial cheek lines (RCL) score ≤3 on the Allergan Fine Lines Scale, who agreed not to change skincare routine or undergo other aesthetic procedures during the study. | <10 days since anticoagulant or anti-inflammatory use, <6 months since botulinum toxin, <1 year since temporary fillers, or if pregnant, lactating, had prior facial surgery, grafts, permanent/semi-permanent fillers, or contraindications per VYC-17.5L label. |
| **Monheit et al. 2018** | USA.  prospective, multicenter, randomized, within subject controlled study. | 123 | Dermatology Clinics / Research Institutes | at 6 months | 5 | yes | NLFSS + FACE-Q +Patient Satisfaction Scales | adults ≥18 years, visible NLFs, severity 2–3 (both sides), agree no other facial treatments below orbital rim | Facial fillers <12 m, fat/Botox <6 m, cosmetic face/neck procedures <6 m, semipermanent fillers/implants, disease/infection/scars in NLF area, hypertrophic scarring, pregnant/nursing, anticoagulant use |
| **Siperstein et al. 2024** | Florida  prospective clinical trial  2020-11-11 to 2023-05-01 | 15 | Private Dermatology Clinic | at 360, 540, and 720 Day. | 4 | yes | NLFSS,NLF,QGSGS, GAIS, AEs, SAEs, Responder Rates, Atrophic Scars, Satisfaction (Investigator and Subject) | adults ≥18 years Good general health, ≥22 years, QGSGS score 4–55. | neuromodulators <6 months, HA fillers <1 year, facial surgery or permanent fillers in the area, allergy to lidocaine/HA, pregnant or nursing, or use of diuretics, antihistamines, chemotherapy, anticoagulants, immunosuppressive/immunomodulatory, or anti-inflammatory drugs <2 weeks before or during the trial. |
| **Sattler et al. 2017** | Germany and the Netherlands.  prospective, open-label, multicenter, observational, post market study.  June 2012 and January 2013. | 70 | Private Dermatology Clinic, Clinical Research Center | at 14 days and 1, 9,12 months | 5 | yes | NLFSS, NLF, QGSGS, GAIS, AEs, SAEs, Responder Rates, Atrophic Scars, Patient and investigators Satisfaction Scales | Male/female ≥18 years, 2 visible symmetrical NLFs, severity 2–3 on 5-point NLFSS, agree to avoid other facial treatments during study | Cosmetic facial procedures altering NLF area, injections in lower face <9 m, semi/permanent fillers or implants, or planning such procedures. |
| **Alimohammadi et al. 2024** | Uppsala, Sweden.  prospective, randomized, double-blind, split-face study.  August 2021 and January 2023. | 48 | Private Dermatology Clinic | at 1, 3, 6, and 9 months. | 5 | Yes | NLF , WSRS, NLF, GAIS, AEs, Responder Rates, Satisfaction, Tolerability | females 18–65 years, no pregnant/ non breast feeding, symmetric NLFs (Grade 3–4 on WSRS). | Porphyria, systemic or wound-healing disorders, recent illness or procedure, severe allergy or lidocaine sensitivity, immune disorders, scars/wounds in area, midface abnormalities or asymmetry, use of coagulation-affecting medications ≤10 days, or recent facial treatments. |

**Table S2.** Industry Sponsorship, Manufacturer Involvement, and Conflict-of-Interest Assessment of the Included Studies

| **Study ID** | **Funding source** | **Manufacturer involvement** | **Author conflicts** | **COI declaration** |
| --- | --- | --- | --- | --- |
| **Xie et al., 2022** | Galderma funded the study and provided study products | Galderma-funded, product supplied by Galderma | All authors are clinical trial investigators for Galderma | Present |
| **Demosthenous et al. 2022** | Allergan Aesthetics, an AbbVie Company | AbbVie employees participated in data interpretation, manuscript review, and the decision to submit; medical writing funded by AbbVie | Demosthenous & Eccleston: Allergan Medical Institute faculty/investigators; Figueiredo & Uva: investigators; Kerson & Silberberg: AbbVie employees | Present, |
| **Del Cueto et al., 2024** | AbbVie | Design, conduct, and financial support by Allergan Aesthetics/AbbVie; AbbVie participated in interpretation, review, and approval; medical writing funded by AbbVie | Del Cueto: none declared; Urdiales Galvez: investigator for AbbVie; Gritti & Kefalas: advisory board/speaker/trainer for AbbVie; de la Guardia & Kerson: AbbVie employees (may hold stock) | Present, |
| **Del Cueto et al., 2024** | AbbVie (same funder/sponsor as above) | Design, conduct, and financial support by Allergan Aesthetics/AbbVie; AbbVie participated in interpretation, review, and approval; medical writing funded by AbbVie | Del Cueto: none declared; Urdiales Galvez: investigator for AbbVie; Gritti & Kefalas: advisory board/speaker/trainer for AbbVie; de la Guardia & Kerson: AbbVie employees (may hold stock) | Present, itemized per author |
| **Dayan et al. 2019** | Allergan plc (Dublin, Ireland) | Allergan plc designed the study and analyzed the data presented in this article." Writing/editorial support (Lela Creutz, PhD, of Peloton Advantage) also funded by Allergan plc | 6 of 8 authors have industry ties: Dayan — researcher/speaker/consultant for Allergan; Maas — researcher/consultant/advisory board for Allergan; Grimes — investigator/advisory board for Allergan, also investigator for Valeant and Galderma; Beer — investigator/consultant/speaker for Allergan, Galderma, Merz, shareholder in Anterios; Monheit — consultant/investigator for Allergan, Revance, Galderma, Merz, Suneva, Teoxane, Alphaeon, CROMA, Evolus, Sienna Pharmaceuticals; Snow — Allergan employee, may own stock; Murphy & Lin — former Allergan employees, employed by Allergan at time of study | Present —. Note: "authors received no honorarium or other form of financial support related to the development of this article" (i.e., no separate authorship payment beyond the disclosed industry relationships) |
| **Rzany et al. 2019** | TEOXANE Laboratories — "Materials and funding for this study were provided by TEOXANE Laboratories" | Study conducted by DERMSCAN (a Contract Research Organization) on TEOXANE's RHA products; TEOXANE staff (F. Bourdon, P. Delobel, K. Legent, S. Meunier, S. Moisenier) are thanked in Acknowledgments for their help — suggesting direct involvement in the trial's conduct/support, though the exact nature of their role (design/analysis/writing) isn't spelled out beyond "materials and funding" and general assistance | Mixed/limited: only 1 of 7 authors discloses a conflict — "Berthold Rzany is a consultant for Merz Aesthetics" (Merz is a *competing* manufacturer, not TEOXANE, the study sponsor). The statement explicitly notes: "The other authors do not report any conflicts" — none of the 7 authors disclose being TEOXANE employees, consultants, or advisory board members despite TEOXANE funding/supplying the study products | Present, |
| **Ogilvie et al. 2019** | Allergan plc (Dublin, Ireland) | Editorial support (Peloton Advantage) funded by Allergan plc; statistical analyses conducted by an Allergan plc employee (Shraddha Mehta, PhD); 3 of 7 authors were Allergan plc employees/stockholders at the time of the study (see below) — study design/analysis role not stated as explicitly as in the AbbVie-era papers, but the sponsor's staff performed the statistics directly | Monheit — investigator for Allergan plc, Galderma, Alphaeon, Teoxane; consultant for Allergan plc, Galderma, Suneva, Merz; Beer — investigator/consultant/speaker for Allergan plc, Galderma, Merz; shareholder in Anterios; partner in The Cosmetic Bootcamp and Theraplex LLC; Grimes — investigator for Allergan plc, Suneva, Alphaeon; consultant for Procter & Gamble; Hardas, Lin, Murphy — were employees and stockholders of Allergan plc at the time of the study; Weichman — provided medical writing assistance funded by Allergan plc | Present — |
| **Monheit et al. 2018** | NO | NO | NO | NO |
| **Siperstein et al. 2024** | Mixed: "Funding was provided by Allergan and Siperstein Dermatology Group" — industry (Allergan) + institutional/practice co-funding | Not detailed beyond funding — no explicit statement about Allergan's role in study design, data analysis, manuscript review, or publication decision (unlike the AbbVie-era papers, which spell this out explicitly). Product (VYC-17.5L/Juvéderm Vollure XC, VYCROSS technology, Allergan Inc.) was used but manufacturer's operational role in the trial is not described | Only 1 of 3 authors discloses a conflict: "R. Siperstein is an Investigator, Consultant, Trainer and Speaker for Allergan/Abbvie and Galderma." Co-authors Nestor and Meran are not mentioned in the disclosure statement at all — their COI status is not explicitly addressed, though the paper states "The authors have indicated no significant interest with commercial supporters" (a somewhat contradictory/ambiguous statement given Siperstein's disclosed industry roles and the Allergan funding line) | Present but inconsistent/ambiguous — the funding line, Siperstein's personal disclosure, and the blanket "no significant interest" statement don't fully reconcile with each other |
| **Sattler et al. 2017** | Allergan plc (Dublin, Ireland) | Not explicitly detailed regarding study design/analysis/publication decision role beyond funding, but manufacturer involvement is evident structurally: 3 of 8 authors (Kolodziejczyk, Kerson, and — per affiliations — others) were Allergan plc employees at the time; 3 more (Van Der Walt, Nathan, Dhillon) were *former* Allergan Ltd. employees; corresponding author (Kolodziejczyk) is an Allergan plc employee with an Allergan email address; "Data on file, Allergan plc" is cited twice as a data source within the manuscript itself | Extensive — of 8 authors: Sattler — lecturer, study investigator, scientific advisory board member for Allergan plc; Philipp-Dormston & Van Den Elzen — lecturers, medical consultants, study investigators, scientific advisory board members for Allergan plc; Van Der Walt, Nathan, Dhillon — former Allergan Ltd. employees; Kolodziejczyk & Kerson — current Allergan plc employees, may own equity. All 8 authors have a disclosed relationship with the manufacturer — no author is free of industry tiesr | Present — |
| **Alimohammadi et al. 2024** | Dr Korman Laboratories Ltd (Kiryat Bialik, Israel) — "The study was sponsored and funded by Dr Korman Laboratories Ltd" | Not explicitly detailed regarding design/analysis/publication-decision role in the manuscript text itself; however, one author (Furman-Assaf) received direct funding from the manufacturer (see below), and the study evaluates the manufacturer's own product (DKL23) against a competitor (Juvéderm Volift) | 2 of 3 authors disclose ties: Furman-Assaf — received funding from Dr Korman Laboratories Ltd (the study sponsor); Alimohammadi — received consulting fees from CTC Clinical Trial Consultants AB (a CRO, not the product manufacturer). Third author (Nilsson) has no disclosed conflict | Present — |

2. **Risk of bias assessment**

**ROBINS-I**

**B**

**A**

**Figure S1. risk of bias assessment by ROBINS-I. (A) distribution for individual studies, (B) the summary of assessment**

**ROB 2**

**B**

**A**

**Figure S2. risk of bias assessment by RoB2. (A) distribution for individual studies, (B) the summary of assessment**

3. **Adverse event:**
   3.1. **BRUISING**

A meta-analysis was performed to pool estimates of the incidence of bruising across six studies. The total pooled incidence of bruising was 54% (95% CI 0.44–0.65), indicating moderate heterogeneity between studies (Tau² = 0.0128; Chi² = 21.89, df = 5, P = 0.0005; I² = 77.2%). Subgroup analysis indicated a significant difference in incidence between investigator-reported and patient-reported bruising (Chi² = 13.89, df = 1, P = 0.0002). The investigator-reported incidence of bruising (1 study, n=53) was 30% (95% CI 0.18–0.44). The patient-reported (5 studies, n=367) pooled incidence of bruising was higher (59%; 95% CI 0.52–0.66) (with low to moderate heterogeneity between studies) (Tau² = 0.0027, Chi² = 6.89, df = 4, P = 0.1418; I² = 42%). Among the patient-reported studies, incidence estimates ranged from 53 to 75%. The highest incidence of bruising was reported by Alimohammadi et al. (2024) (75%; 95% CI 0.60–0.86), and the lowest by Siperstein et al. (2022) and Demosthenous et al. (2022) (53%; 95% CI 0.27–0.79 and 0.40–0.66, respectively), with Dayan et al. (2019) and Monheit et al. (2018) both reporting an incidence of bruising of 57% (95% CI 0.47–0.66) **Figure (S3).**

**Figure S3.** Forest plot of the bruising

3.2. **Discoloration.**

Pooled incidence of discoloration calculated from the meta-analysis of three studies was 27% (95% CI 0.22–0.32). There was no heterogeneity among included studies (I² = 0%, P = 0.975). Individual study estimates were highly consistent. Demosthenous et al. (2022) reported an incidence of 28% (95% CI 0.17–0.41), while Dayan et al. (2019) and Monheit et al. (2018) both reported an incidence of 27% (95% CI 0.19–0.36). No subgroup analysis was obtained, as there was only one assessment source (investigator reported) across all studies **Figure (S4).**

**Figure S4.** Forest plot of the discoloration

3.3. **induration**

Meta-analysis of induration showed substantial and statistically significant differences by source of assessment (test for subgroup differences: P < 0.0001). When induration was investigator-reported (2 studies, n = 228), the pooled incidence was extremely low at 1% (95% CI 0.00–0.08), with significant heterogeneity between the two studies (I² = 80.8%). Ogilvie et al. reported a 4% incidence (95% CI 0.00–0.13), while Xie et al. reported a 0% incidence (95% CI 0.00–0.02). When induration was patient-reported (3 studies, n = 304), the pooled incidence was 88% (95% CI 0.84–0.92), with no heterogeneity between the studies (I² = 0%). Estimates from the three studies pooled within this group were extremely similar: Demosthenous et al. reported 87% (95% CI 0.75–0.94), while Dayan et al. and Monheit et al. were both 89% (95% CI 0.81–0.94). Due to the extreme differences between subgroups, the pooled incidence of 49% (95% CI 0.04–0.95) across all five studies was not interpretable and was characterized by near-total heterogeneity (I² = 99.4%, P < 0.0001) **Figure (S5).**

**Figure S5.** Forest plot of the induration

3.4. **Itching**

The meta-analysis showed a highly significant difference in reported incidence of itching by source of assessment (test for subgroup differences: P < 0.0001). When itching was reported to the investigator (3 studies, n = 216), the pooled incidence was 1% (95% CI 0.00–0.04). There was no heterogeneity among studies (I² = 0%). Individual study estimates within this subgroup were 1% (Nasuti et al.) to 2% (Lendvai et al.). When itching was patient-reported (5 studies, n = 367), the pooled incidence was 28% (95% CI 0.23–0.33). There was no heterogeneity among studies (I² = 0%). Study-specific estimates within this subgroup ranged from 20% (Demosthenous et al.) to 31% (Dayan et al. and Monheit et al.). Due to the large discrepancy between these subgroups, the overall pooled incidence across all eight studies (10%, 95% CI 0.00–0.49) is not clinically meaningful and is highly heterogeneous (I² = 93.0%, P < 0.0001) **Figure (S6).**

**Figure S6.** Forest plot of the itching

3.5. **lumps sensitivity analysis**

The primary meta-analysis showed a highly significant difference in the reported incidence of lumps by source of assessment (test for subgroup differences: P < 0.0001). Due to this extreme difference, a sensitivity analysis was conducted. When lumps were investigator-reported (three studies, n = 338), pooled incidence was 1% (95% CI 0.00–0.03) with low heterogeneity (I² = 14.4%). Estimates for this subgroup were all low and similar, ranging from 1% to 3%. When lumps were patient-reported (three studies, n = 304), pooled incidence was 81% (95% CI 0.76–0.85) with no heterogeneity (I² = 0%). Estimates for this subgroup were all high and similar, ranging from 75% to 82%. Given the striking difference between the results of these two subgroups, the overall pooled incidence across all six studies (41%, 95% CI 0.06–0.85) is uninformative and not clinically interpretable, with near-total heterogeneity (I² = 99.2%, P < 0.0001). The sensitivity analysis confirms that the reported incidence of lumps is not one estimate but entirely dependent on the method of assessment **Figure (S7).**

**Figure S7.** Forest plot of the lumps sensitivity analysis.

3.6. **Pain after injection sensitivity**

The meta-analysis showed a statistically significant and clinically important difference in the reported incidence of post-injection pain according to the source of assessment (test for subgroup difference: P < 0.0001). When pain was assessed by investigators (3 studies, n = 216), the pooled incidence was 7% (95% CI 4–11%) with no heterogeneity across studies (I² = 0%). Study-specific estimates within this subgroup were all low, ranging from 4% to 8%. When pain was patient-reported (4 studies, n = 319), the pooled incidence was much higher at 71% (95% CI 64–77%), with low heterogeneity (I² = 23.3%). Study-specific estimates ranged from 47% to 72%. Because of the large difference between subgroups, the overall pooled incidence across all seven studies (37%, 95% CI 12–66%) is not clinically interpretable and shows extreme heterogeneity (I² = 97.8%, P < 0.0001). This sensitivity analysis confirmed that the incidence of post-injection pain was highly dependent on the method of assessment, with patient-reported rates of post-injection pain about tenfold higher than those reported by investigators **Figure (S8).**

**Figure S8.** Forest plot of the Pain after injection sensitivity analysis.

3.7. **redness sensitivity**

Meta-analysis of the incidence of redness by source of assessment showed that this was statistically and clinically significantly different between subgroups (test for subgroup differences, P < 0.0001). When redness was assessed by the investigator (2 studies, n = 258), the pooled incidence was 1% (95% CI 0–2%) and was free from heterogeneity between studies (I² = 0%). Pooled and study-specific point estimates were all at 1%. In contrast, when the redness was self-assessed by the patient (5 studies, n = 367), the pooled incidence was substantially higher at 73% (95% CI 68–77%) and was free from heterogeneity between studies (I² = 0%) with study-specific estimates ranging from 60% to 74%. Owing to the extreme subgroup difference in estimates, the pooled incidence across all 7 studies (37%, 95% CI 8–77%) was not clinically interpretable and was highly heterogeneous (I² = 98.8%, P < 0.0001). This sensitivity analysis therefore confirmed that the incidence of redness is highly dependent on the method of assessment, with patient-reporting being over 70 times higher than investigator-reported **Figure (S9).**

**Figure S9.** Forest plot of the redness sensitivity

3.8. **swelling sensitivity analysis**

The meta-analysis demonstrated a highly significant and clinically relevant difference in the reported incidence of swelling by source of assessment (test for subgroup differences, P < 0.0001). When swelling was assessed by investigators (3 studies, n = 311), the pooled incidence was 1% (95% CI 0–6%) with moderate heterogeneity across studies (I² = 62.7%, P = 0.0685). The point estimates in each study were low, ranging from 0% to 2%. When swelling was assessed by the patient (4 studies, n = 352), the pooled incidence was 87% (95% CI 83–91%) with low heterogeneity (I² = 21.7%, P = 0.2801). The point estimates in each study were high, ranging from 81% to 93%. Because of this extreme difference between the subgroups, the overall pooled incidence of swelling across all seven studies, 43% (95% CI 4–90%), is clinically meaningless and is highly heterogeneous (I² = 99.3%, P < 0.0001). This sensitivity analysis confirms that the reported incidence of swelling is highly dependent on the method of assessment, with a patient-reported incidence that is approximately 87 times greater than the incidence of swelling assessed by investigators **Figure (S10).**

**Figure S10.** Forest plot of the swelling sensitivity analysis

3.9. **Tenderness to touch sensitivity analysis**

The meta-analysis revealed a statistically significant and clinically substantial difference in the reported incidence of tenderness to touch according to the source of assessment (test for subgroup differences: P < 0.0001). When tenderness was assessed by investigators (2 studies, n = 258), the pooled incidence was 0% (95% CI 0–2%), with moderate heterogeneity across studies (I² = 50.4%). Individual study estimates were 0% and 1%. In contrast, when tenderness was patient-reported (4 studies, n = 319), the pooled incidence was markedly higher at 82% (95% CI 75–88%), with moderate heterogeneity (I² = 44.5%). Study-specific estimates ranged from 60% to 84%. Owing to this extreme divergence between subgroups, the overall pooled incidence across all six studies (45%, 95% CI 6–89%) lacks clinical interpretability and exhibits extreme heterogeneity (I² = 99.2%, P < 0.0001). This sensitivity analysis confirms that the reported incidence of tenderness to touch is profoundly influenced by the assessment method, with patient-reported rates being dramatically higher than those reported by investigators **Figure (S11).**

**Figure S11.** Forest plot of the Tenderness to touch sensitivity analysis
